# A Phase 1, open-label, multicentre study to compare the capsule and tablet formulations of AZD5363 and explore the effect of food on the pharmacokinetic exposure, safety and tolerability of AZD5363 in patients with advanced solid malignancies: OAK

**DOI:** 10.1007/s00280-018-3558-z

**Published:** 2018-03-14

**Authors:** Emma Dean, Udai Banerji, Jan H. M. Schellens, Matthew G. Krebs, Begona Jimenez, Emilie van Brummelen, Chris Bailey, Ed Casson, Diana Cripps, Marie Cullberg, Stephen Evans, Andrew Foxley, Justin Lindemann, Paul Rugman, Nigel Taylor, Guy Turner, James Yates, Peter Lawrence

**Affiliations:** 1 The Christie NHS Foundation Trust, The University of Manchester, Manchester, UK; 2 Royal Marsden Hospital, London, UK; 3 Netherlands Cancer Institute, Amsterdam, The Netherlands; 4 IMED Biotech Unit, AstraZeneca, Cambridge, UK; 5 AstraZeneca, Macclesfield, UK; 6 AstraZeneca, Da Vinci Building, Melbourn Science Park, Melbourn, Hertfordshire SG8 6HB UK

**Keywords:** AZD5363, AKT inhibitor, Capsule, Tablet, Fed:fasted, Advanced solid tumour

## Abstract

**Purpose:**

AZD5363 is a potent pan-AKT inhibitor originally formulated as a capsule; a tablet was developed for patient convenience and manufacturing ease. This study assessed the PK comparability of both formulations (Part A) and the effect of food (Part B) on the PK/safety of the tablet.

**Methods:**

Adults with advanced solid tumours received AZD5363 480 mg bid in a partially fasted state by tablet (Week 1) and capsule (Week 2) in a ‘4-days-on/3-days-off’ schedule (Part A). PK parameters were evaluated using pre-defined 90% CIs for AUCτ and *C*_max_ ratios of 0.75–1.33 to assess comparability. In Part B, AZD5363 tablet was given to a new cohort of patients under the same conditions as Part A, except on the morning of PK assessment days, when it was administered after an overnight fast (Week 1) and standard meal (Week 2).

**Results:**

In evaluable patients (*N* = 11), the geometric least-squares mean ratios (tablet:capsule) for AUCτ and *C*_max_ were 0.90 (0.77–1.06) and 1.02 (0.86–1.20), respectively, demonstrating comparable PK in the partially fasted state. Tablet and capsule safety data were also comparable. Tablet PK profiles indicated later t_max_ and lower *C*_max_ after food versus overnight fast. Fed and fasted AUCτ and *C*_max_ ratios were 0.89 (0.76–1.05) and 0.67 (0.55–0.82), respectively (*N* = 9). The safety/tolerability profile of the tablet was comparable between fed and fasted states.

**Conclusions:**

PK and safety/tolerability of AZD5363 tablet and capsule were comparable. Food did not affect the bioavailability of AZD5363, but reduced the absorption rate without discernibly affecting safety/tolerability.

## Introduction

The phosphoinositide 3-kinase/serine–threonine protein kinase AKT/mammalian target of rapamycin (PI3K/AKT/mTOR) signalling pathway is frequently deregulated in human cancer [1] and is therefore a promising target for the development of new therapies [2, 3]. As a key component of the signalling network that mediates processes such as cell proliferation and resistance to apoptosis, AKT (expressed in three isoforms: AKT1, 2 and 3) has been shown to be overexpressed or activated in a wide range of solid and haematological malignancies [3, 4]. Activation of all three isoforms of AKT is associated with anticancer drug resistance, advanced disease and poor prognosis [5]. Several diverse small molecule compounds, such as ATP-competitive inhibitors, phosphatidylinositol analogues and allosteric inhibitors, have been developed to block the AKT pathway, but while effective inhibition has been shown at nanomolar concentrations, non-selective targeting of other protein kinases (including PKA and PKC) limits the therapeutic utility of some treatments [3].

AZD5363 is a potent, selective inhibitor of AKT1, 2 and 3 [6, 7]. Preclinical studies of single and chronic oral dosing of AZD5363 in mice have demonstrated dose-dependent inhibition of AKT substrate phosphorylation (GSK3β and PRAS40), tumour cell proliferation, and tumour growth in xenograft models [6]. Furthermore, increasing evidence supports the antitumour efficacy of AZD5363 in overcoming or delaying resistance to hormone therapy, chemotherapy or HER2-directed therapies in patient-derived gastric, breast and prostate cancer xenograft models [6, 8–10]. Antitumour activity of AZD5363, established across a range of preclinical models, has been reported in Phase 1, open-label clinical studies in patients with solid tumours as monotherapy and in combination, which were conducted with the primary objectives of evaluating the safety, tolerability, pharmacokinetic (PK) and pharmacodynamic (PD) effects of AZD5363 under adaptable dosing schedules [11–14]. From these studies, recommended Phase 2 doses were established for AZD5363 monotherapy in a ‘4-days-on/3-days-off’ schedule (4/3 schedule; 480 mg twice daily [bid]), as well as a ‘2-days-on/5-days-off’ schedule (2/5 schedule; 640 mg bid) [11, 13, 14]. With the 4/3 schedule, PK results were comparable to exposures associated with tumour regression in preclinical models. AZD5363 plasma exposure was approximately dose proportional in the dose range 80–800 mg and terminal half life was approximately 10 h [11]. The major elimination route is assumed to be hepatic, as less than 10% of the dose was excreted in urine as unchanged drug. The observed adverse events (AEs) of diarrhoea, rash and hypersensitivity, and hyperglycaemia were consistent with the effects of AKT inhibition [11, 13, 14].

AZD5363 was administered to patients in the early clinical trial programme as a capsule formulation in a partially fasted state (no caloric intake from 2 h before dosing to 1 h after dosing). However, for patient convenience and ease of manufacturing, a tablet formulation has now been developed. Before widespread introduction of the tablet formulation into the clinical programme, PK comparability with the capsules needed to be verified. Furthermore, an investigation of the effect of food and an overnight fast would inform dosing recommendations. Consequently, this two-part Phase 1 study was performed to determine whether the capsule and tablet formulations of AZD5363 are comparable in terms of PK exposure to compare the safety and tolerability profiles of the two formulations (Part A), and to make a preliminary assessment of the effect of food on the PK, safety and tolerability of the tablet formulation (Part B).

## Methods

### Study design

This was an open-label, multicentre, two-part, fixed-sequence, crossover study of AZD5363 in adult patients with advanced solid malignancies, which was conducted between December 2013 and July 2015 at two sites in the UK and one in the Netherlands (ClinicalTrials.gov NCT01895946; AstraZeneca study name, OAK). The trial was performed in accordance with the principles of the Declaration of Helsinki, Good Clinical Practice and the AstraZeneca policy on bioethics [15]. The local ethics committee or independent review board at each investigator site approved the protocol prior to study commencement. All patients provided written informed consent prior to study participation.

In Part A, AZD5363 tablet (Week 1) and capsule (Week 2) forms were given orally at 480 mg bid in a 4/3 schedule (i.e. 4 days on treatment, 3 days off treatment). AZD5363 tablet or capsule was taken at approximately the same time in the morning and evening, in a partially fasted state (from 2 h before until 1 h after each dose). PK parameters were assessed on the fourth day of Week 1 and Week 2 (i.e. on Day 4 and Day 11).

In Part B, which occurred following completion of Part A, a new cohort of patients received the AZD5363 tablet at the same dose and schedule as in Part A. In Week 1, AZD5363 tablet was administered in a partially fasted state except on PK assessment Day 4, when fasting was overnight (minimum of 8 h) until 4 h post-dose. In Week 2, AZD5363 tablet was administered in a partially fasted state, except on PK assessment Day 11 in Week 2, when the morning dose was given in a fed state. The tablet was administered 30 min after starting a standardized meal containing 605 kcal, of which 24% (36 g) were from proteins, 26% (18 g) from fat and 50% (76 g) from carbohydrates. Patients had been fasting overnight prior to the breakfast and no further food was permitted for 4 h post-dose.

From Week 3 onwards, patients received AZD5363 capsule in Part A and AZD5363 tablet in Part B. Both formulations were administered according to the standard dietary restrictions for the study treatment (i.e. partially fasted).

In Part A, an initial cohort of six patients was enrolled. If this cohort was insufficient to establish comparability of dose or PK exposure of the AZD5363 tablet and capsule, additional patients could be enrolled (up to a maximum of 24 patients in total) to further evaluate comparability. In Part B, an initial cohort of six patients was enrolled, followed by possible further recruitment of up to a maximum of 12 patients to explore the effect of food on tablet formulation exposure. Cohort expansion, dosing adjustment of the tablet formulation, or exploration of changes in the dose schedule was considered after review of safety and tolerability and PK data by the scientific review committee. For both Parts A and B, patients received AZD5363 for as long as they continued to show clinical benefit, as judged by the investigator, and in the absence of disease- or treatment-related discontinuation criteria.

### Patient selection

Eligible patients were aged ≥ 18 years and had a confirmed (histologically or, where appropriate, cytologically) malignant solid tumour refractory or resistant to standard therapy or for which no suitable standard therapy existed. Patients were required to have a World Health Organization (WHO) performance status of 0 or 1, with no clinical deterioration in the previous 2 weeks and a life expectancy of at least 12 weeks. Willingness to fast and to eat the standardized meal was a further requirement for entry into Part B of the study.

Exclusion criteria included treatment with chemotherapy, immunotherapy, anticancer agents, and specific CYP3A4 inducers/inhibitors/substrates or CYP2D6 substrates, or nitrosourea or mitomycin C up to 6 weeks prior to commencing study treatment; major surgery or radiotherapy within 4 weeks of initiation of study treatment; clinically significant glucose metabolic abnormalities; severe or uncontrolled systemic disease; pre-specified cardiac conditions; inadequate bone marrow reserve or organ function; and hypersensitivity to AZD5363 or drugs with similar chemical structure. In addition, patients with a significant gastrointestinal condition that precluded fasting or ingestion of the standardized meal were excluded from Part B.

### Study objectives

Co-primary objectives of the study were to assess the PK exposure of the AZD5363 tablet and the capsule (Part A) and to investigate the effect of a standardized meal on the PK exposure of the AZD5363 tablet (Part B). Secondary objectives included to generate multiple-dose PK data for the AZD5363 tablet and capsule (Part A) and for the AZD5363 tablet in the presence/absence of food (Part B); and to explore the safety/tolerability of the AZD5363 tablet and capsule (Part A) and the effect of food on the safety/tolerability of the AZD5363 tablet (Part B).

### Assessments

PK profiles of AZD5363 tablet and capsule formulations were characterized from venous blood samples drawn pre-dose and 0.5, 1, 2, 4, 6, 8 and 12 h after the morning dose on Day 4 and Day 11 in Parts A and B. In addition, pre-dose samples were drawn on Day 8 in Part B to verify that a washout period of 3.5 days was adequate for AZD5363.

The bioanalytical method used to generate AZD5363 plasma concentration data for the derivation of PK parameters for the study had been previously validated, according to regulatory guidance [16, 17], by Covance UK Ltd on behalf of AstraZeneca. Following the addition of internal standard ([^13^C_6_]AZ12886380), calibration, quality control (QC) and study samples (25 µL) were processed by solid-phase extraction then assayed for AZD5363 by liquid chromatography–tandem mass spectrometry. The lower limit of quantification of the assay was 1.00 ng/mL, with the assay demonstrating linearity up to 1000 ng/mL. Although the majority (> 67%) of samples assayed had concentrations within the validated assay range, any samples found or expected to have concentrations in excess of the validated assay range were appropriately diluted in control plasma prior to extraction and re-assayed/assayed with dilution QC samples included to validate the dilution scheme on a batch-by-batch basis. All trial samples were analysed within the known stability period for AZD5363 and all a priori assay acceptance criteria were met, in accordance with regulatory guidance.

Primary PK parameters assessed included the area under the plasma concentration–time curve during the 0- to 12-h dosing interval (AUCτ), maximum concentration (*C*_max_), minimum concentration (*C*_min_), and time to maximum concentration (*t*_max_). PK parameters were derived using non-compartmental methods (Phoenix^®^ WinNonlin^®^). Plasma concentration–time profiles were analysed to determine *C*_max_, *C*_min_ and *t*_max_, while AUCτ was determined from application of the linear-up/log-down trapezoidal rule.

Safety assessments throughout the study encompassed the incidence and severity of AEs (graded according to Common Terminology Criteria for Adverse Events [CTCAE] version 4.0), in addition to laboratory parameters (haematology, clinical chemistry, urinalysis), physical examination, 12-lead electrocardiography (ECG), and vital signs. To assess safety in the fed versus fasted state, the period during and just after the fasted day was considered relevant to the fasted dose (Days 4–7), and similarly, the fed day and subsequent days were considered relevant to the fed state (Days 11–14). Any AE occurring after the first dose of study treatment and up to 28 days after the last dose of study treatment was included in the AE summaries.

### Statistical analysis

Sample size calculations for PK assessment during Part A were based on the statistical assumption that within-patient mean squared error (MSE) for AUCτ was 0.0289 (log scale), with standard deviation (SD) differences of 0.241. Similarly, the within-patient MSE calculation for *C*_max_ was assumed to be 0.0484 (log scale), with SD differences of 0.311. Based on these assumptions, and a type I error of 5% (for each hypothesis), 12 patients were judged sufficient to demonstrate with at least 80% power that the 90% confidence intervals (CIs) for AUCτ and *C*_max_ would fall entirely within the equivalence boundaries of 0.75–1.33, which was considered suitable to demonstrate comparability of the two formulations in Part A. No formal sample size calculations were performed for Part B, although 12 patients were considered appropriate to provide an estimate of the food effect.

A patient was included in the PK analysis set if they had taken the drug as required by the protocol on Day 4 and Day 11 of PK sampling and on the previous 2 days (Part A and B), had a full set of PK samples for both tablet and capsule (Part A) or for both fasted and fed states (Part B), had fasted overnight according to the protocol (Part B) and consumed most of the standardized meal within the 30-min time frame on Day 11 (Part B).

The statistical analysis for estimating the food and capsule effect was based on analysing log-transformed PK parameters AUCτ and *C*_max_ using a fixed-effects model with terms for patient and treatment only. All statistical calculations and analyses were performed with SAS^®^ software, version 9.1.3 or higher. Other PK parameters are presented descriptively.

The safety analysis set comprised all patients who received at least one dose of AZD5363. All safety data are presented descriptively.

## Results

### Patients

Overall, 18 patients with advanced solid tumours were enrolled and received AZD5363 in Part A. Based on the results of Part A, the AZD5363 tablet at a dose of 480 mg bid in a 4/3 schedule was administered to 12 patients in Part B. Baseline demographic and clinical characteristics of the enrolled population are summarized in Table 1. All patients had metastatic disease. In Part A, the majority of patients (10/18, 56%) had primary tumours of gastrointestinal origin (colon, *n* = 2; colorectal, *n* = 3; small bowel, *n* = 1; and rectal, *n* = 4).

**Table 1:** Patient demographics and baseline characteristics in Parts A and B

	Patients			
	PK set		Full set	
	Part A (*N* = 11)	Part B (*N* = 9)	Part A (*N* = 18)	Part B (*N* = 12)
Median age (range), years	61 (48–70)	63 (42–75)	60 (45–70)	62 (42–75)
Male:female, *n:n*	6:5	5:4	10:8	5:7
Race, *n* (%)				
White	10 (90.9)	9 (100)	17 (94.4)	11 (91.7)
Other	1 (9.1)	0	1 (5.6)	1 (8.3)
Median weight (range), kg	77 (53–115)	81 (65–96)	78 (53–115)	80 (54–96)
WHO performance status, *n* (%)				
0	5 (45.5)	2 (22.2)	6 (33.3)	2 (16.7)
1	6 (54.5)	7 (77.8)	12 (66.7)	10 (83.3)
Extent of disease, *n* (%)				
Metastatic	11 (100)	9 (100)	18 (100)	12 (100)
Primary tumour site, *n* (%)				
Gastrointestinal^a^	7 (63.6)	2 (22.2)	10 (55.6)	3 (25.0)
Lung	0	4 (44.4)	1 (5.6)	4 (33.3)
Breast	0	2 (22.2)	1 (5.6)	2 (16.7)
Other^b^	4 (36.4)	1 (11.1)	6 (33.3)	3 (25.0)
Prior lines of chemotherapy^c^, *n* (%)				
0	0	0	0	0
1	2 (18.2)	2 (22.2)	3 (16.7)	2 (16.7)
≥ 2	9 (81.8)	7 (77.8)	14 (77.8)	9 (75.0)

^a^Includes colon, colorectal, small bowel, and rectal

^b^Includes adrenal, bladder, liver, other, prostate

^c^Information not available for one patient in the full set in each part

### Pharmacokinetics

**Fig. 1:**
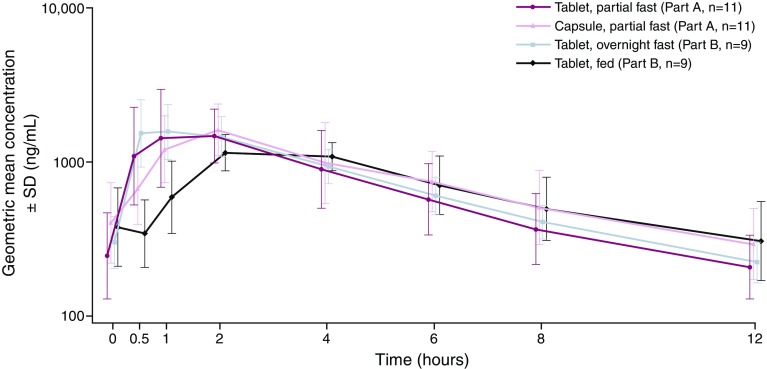
Geometric mean (± SD) plasma concentration–time profiles for the AZD5363 tablet and capsule given partially fasted (Part A), and the tablet given after an overnight fast and food (Part B). Summarizes plasma concentrations by nominal sample time, and the symbols have been staggered for clarity of presentation. Geometric mean (± SD) is calculated as exp(µ ± s), where µ and s are the mean and standard deviation of the plasma concentrations on the log_e_ scale

**Fig. 2:**
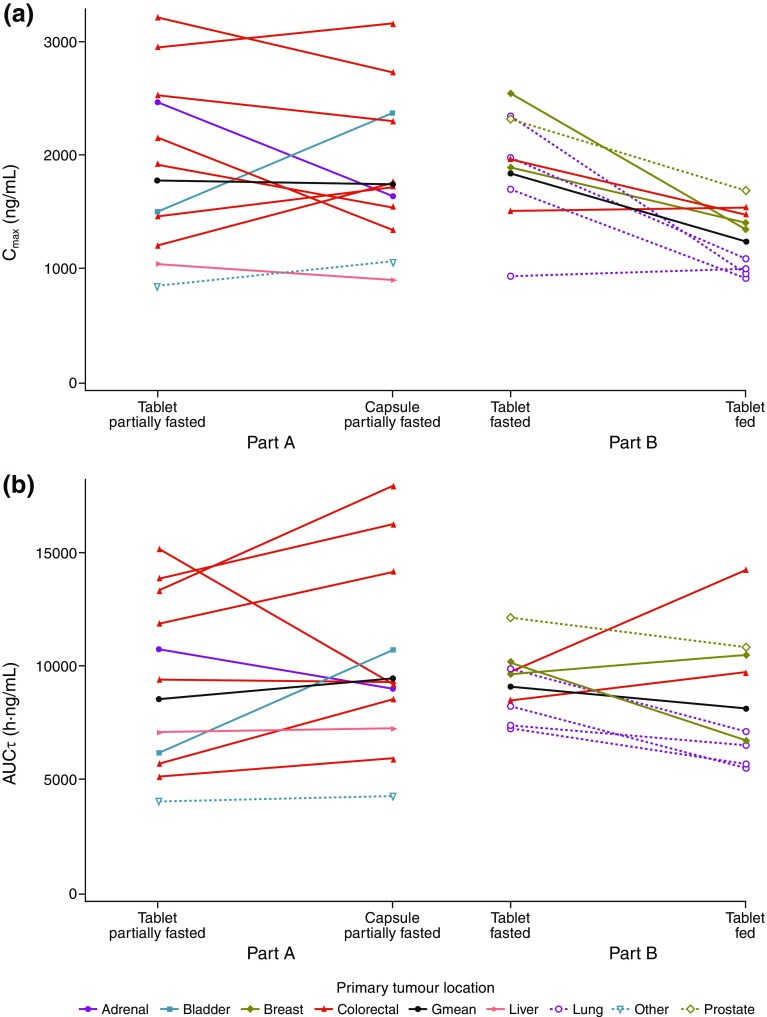
Matched pairs of the PK parameters AUCτ and *C*_max_ for **a** tablet versus capsule in the partially fasted state (Part A) and **b** tablet in the fasted versus fed state (Part B). *Gmean* geometric mean

Geometric mean plasma concentration–time profiles from both study parts (Parts A and B) are shown in Fig. 1 and individual PK parameters (AUCτ and *C*_max_) in Fig. 2. The primary tumour location did not appear to influence the systemic exposure to AZD5363.

#### Part A

Overall, 11 patients were evaluable for PK analysis in Part A; insufficient amount of study drug was taken by two patients, four patients did not provide sufficient samples for PK analysis, and one patient did not have their PK samples collected according to the protocol time points.

The geometric mean AZD5363 plasma concentration–time profiles indicated faster absorption from the tablet than from the capsule (Fig. 1), supported by the shorter observed median times to maximum plasma concentration, *t*_max_ (Table 2). The geometric means for AUCτ and *C*_max_ at steady state were, however, similar between the AZD5363 tablet and capsule, and the 90% CIs for the tablet and capsule ratios of the geometric least-squares mean fell within the pre-defined boundaries (0.75–1.33), demonstrating comparable exposure (Table 2). The inter-patient variability (coefficient of variation) in AUCτ was 45 and 48% for the capsule and tablet, respectively; the corresponding values for *C*_max_ were 40 and 46%. Intra-patient variability was estimated to be 20% for AUCτ and 22% for *C*_max_.

**Table 2:** PK parameters for steady-state AZD5363 in Parts A and B (PK analysis set)

Parameter	*N*	Part A			
		Tablet	Capsule	Ratio tablet:capsule	
		Gmean (CV%)	Gmean (CV%)	GLSM ratio	90% CI
AUCτ, h ng/mL	11	8536 (47.7)	9445 (44.6)	0.90	0.77, 1.06
*C*_max_, ng/mL	11	1779 (46.2)	1745 (40.2)	1.02	0.86, 1.20
*C*_min_, ng/mL	11	192.6 (55.6)	271.5 (57.7)	NC	NC
*t*_max_, h^a^	11	1.0 (0.6–2)	2.0 (1–4)		

Parameter	*N*	Part B			
		Fasted	Fed	Ratio fed:fasted	
		Gmean (CV%)	Gmean (CV%)	GLSM ratio	90% CI
AUCτ, h ng/mL	9	9116 (16.8)	8136 (33.9)	0.89	0.76, 1.05
*C*_max_, ng/mL	9	1842 (31.1)	1239 (23.3)	0.67	0.55, 0.82
*C*_min_, ng/mL	9	218.5 (30.3)	262.3 (58.6)	NC	NC
*t*_max_, h^a^	9	0.6 (0.5–4)	2.0 (2–4.3)		

*CV* coefficient of variation, *GLSM* geometric least-squares mean, *Gmean* geometric mean, *NC* not calculated

^a^Median (range)

#### Part B

Of the 12 patients who received treatment in Part B, nine were included in the PK analysis population; insufficient amount of study drug was taken by one patient, one patient did not consume the majority of the standard meal, and one patient did not provide sufficient samples for PK analysis.

The geometric mean AZD5363 plasma concentration–time profiles indicated a time lag in the absorption and a lower absorption rate when the tablet was given with food, compared with after an overnight fast (Fig. 1). This resulted in lower and later peak concentrations (Table 2). For steady-state *C*_max_, the lower bound of the 90% CI for the geometric least-squares mean ratio was outside the boundaries of 0.75–1.33, but the steady-state AUCτ ratio demonstrated comparable extent of exposure (Table 2). The inter-patient variability (coefficient of variation) in AUCτ was 17 and 34% for the fasted and fed administrations, respectively; the corresponding values for *C*_max_ were 31 and 23%. Intra-patient variability was estimated to be 19% for AUCτ and 23% for *C*_max_.

Samples collected before dosing on Day 1 contained no quantifiable AZD5363. Samples collected pre-dose on Day 8 (i.e. 3 days after the last dose on Day 4) contained low concentrations (geometric least-squares mean 3.34 ng/mL) compared with the average steady-state concentration over the 12-h dosing interval on Day 11 (geometric mean AUCτ/12 = 8136/12 = 678 ng/mL).

### Safety and tolerability

#### Part A

Eighteen patients who received AZD5363 in Part A were included in the safety analysis population. The most commonly reported AEs, irrespective of causal relationship to AZD5363 treatment, were diarrhoea, hyperglycaemia, and nausea (Table 3). Only one grade 4 AE was observed during the study, which was an event of hypokalaemia with onset on Day 16. Hyperglycaemia was the most common overall grade 3 AE observed in six patients in the Day 1–7 time frame (Table 3).

**Table 3:** AEs observed in Part A occurring in ≥ 2 patients in either of the first 2 weeks or of grade ≥ 3 severity (safety population)

Number of patients (%)	Tablet (*N* = 18) Days 1–7		Capsule (*N* = 18) Days 8–14		Capsule (*N* = 18) Day 15 onward^a^	
	All	Grade ≥ 3	All	Grade ≥ 3	All	Grade ≥ 3
Any AE (irrespective of causality)	17 (94.4)	8 (44.4)	15 (83.3)	4 (22.2)	16 (88.9)	7 (38.9)
Any AE (causally related^b^)	17 (94.4)	7 (38.9)	12 (66.7)	4 (22.2)	14 (77.8)	6 (33.3)
AE by preferred term (irrespective of causality)						
Diarrhoea	11 (61.1)	1 (5.6)	6 (33.3)	0	9 (50.0)	5 (27.8)
Hyperglycaemia	7 (38.9)	6 (33.3)	4 (22.2)	1 (5.6)	2 (11.1)	1 (5.6)
Nausea	4 (22.2)	0	2 (11.1)	0	3 (16.7)	0
Anaemia	1 (5.6)	0	3 (16.7)	0	0	0
Pyrexia	0	0	3 (16.7)	0	0	0
Rash maculopapular	0	0	2 (11.1)	2 (11.1)	0	0
Fatigue	3 (16.7)	0	0	0	4 (22.2)	2 (11.1)
Constipation	2 (11.1)	0	0	0	1 (5.6)	0
Vomiting	2 (11.1)	0	0	0	5 (27.8)	0
Tumour pain	1 (5.6)	1 (5.6)	0	0	0	0
Blood bilirubin increased	0	0	2 (11.1)	0	0	0
ECG QT prolonged	0	0	2 (11.1)	0	0	0
Hypokalaemia	1 (5.6)	0	1 (5.6)	0	2 (11.1)	1 (5.6)
Hyponatraemia	0	0	1 (5.6)	1 (5.6)	0	0
Dyspnoea	0	0	0	0	2 (11.1)	1 (5.6)
Pulmonary embolism	0	0	0	0	1 (5.6)	1 (5.6)
Intestinal obstruction	0	0	0	0	1 (5.6)	1 (5.6)
Rash macular	0	0	0	0	1 (5.6)	1 (5.6)
Rash papular	0	0	1 (5.6)	1 (5.6)	0	0

^a^AEs with onset from Day 15 up to 28 days after the date of the last dose of AZD5363

^b^As assessed by the investigator

Nine serious AEs (SAEs) were observed in 7 of 18 patients (38.9%) in Part A. In two of these patients, the events occurred prior to the start of study treatment. SAEs of mouth haemorrhage, papular rash, and pyrexia were each reported in one patient during treatment, and diarrhoea, vomiting, pulmonary embolus, and intestinal obstruction were each reported by one patient in the follow-up phase. Of these SAEs, only papular rash and diarrhoea were considered to be related to treatment, and only papular rash led to treatment discontinuation. Two other patients discontinued treatment because of AEs (nausea, macular rash). Five patients had study treatment interruptions as a result of AEs during the course of the study, and median relative dose intensity was 100% (quartile 1, 94.0%; quartile 3, 100%).

**Fig. 3:**
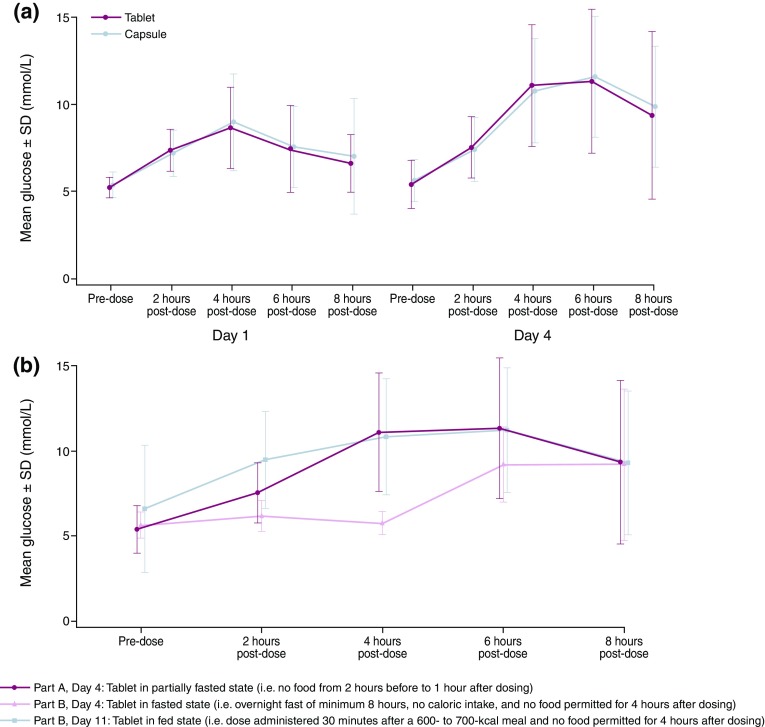
**a** Glucose profiles following AZD5363 capsule or tablet dosing in a partially fasted state (Part A). **b** Plasma glucose profiles following AZD5363 tablet dosing in the fed, partially fasted, and fasted states

There were no clinically significant changes in ECG findings, vital signs or physical examinations and no clinically important, treatment-related trends in clinical chemistry parameters. Elevations in plasma glucose (with associated increases in plasma insulin and C-peptide) were observed in the majority of patients dosed with AZD5363. The profile of these elevations appears to be similar for the tablet and capsule formulations (Fig. 3a).

#### Part B

The most common AEs experienced by patients in Part B are summarized in Table 4. Six patients experienced 11 SAEs, which were diarrhoea (*n* = 3), maculopapular rash (*n* = 2), pneumonia, hyperglycaemia, dyspnoea, stomatitis, vomiting, and papular rash (each *n* = 1). In four patients, the SAEs were considered related to AZD5363: diarrhoea (*n* = 2), maculopapular rash (*n* = 2), stomatitis, and papular rash (both *n* = 1). Three patients discontinued AZD5363 because of AEs, which were pneumonia, maculopapular rash, and papular rash (each *n* = 1). Six patients had dose interruptions, which were a result of AEs in five patients. Overall, median relative dose intensity was 96.2% (quartile 1, 70.6%; quartile 3, 99.3%). There were no clinically important, treatment-related trends in clinical chemistry parameters.

**Table 4:** AEs observed in Part B occurring in ≥ 2 patients in either of the two initial treatment periods, or of grade ≥ 3 severity (safety population)

Number of patients (%)	Days 4–7, fasted dose (*N* = 12)		Days 11–14, fed dose (*N* = 10)^a^		Day 15 onward^b^, partially fasted (*N* = 12)	
	All	Grade ≥ 3	All	Grade ≥ 3	All	Grade ≥ 3
Any AE (irrespective of causality)	5 (41.7)	2 (16.7)	6 (60.0)	4 (40.0)	12 (100.0)	7 (58.3)
Any AE(causally related^c^)	4 (33.3)	2 (16.7)	5 (50.0)	4 (40.0)	10 (83.3)	5 (41.7)
AE by preferred term (irrespective of causality)						
Diarrhoea	2 (16.7)	1 (8.3)	3 (30.0)	2 (20.0)	8 (66.7)	1 (8.3)
Hyperglycaemia	1 (8.3)	1 (8.3)	1 (10.0)	1 (10.0)	6 (50.0)	5 (41.7)
Rash maculopapular	1 (8.3)	0	2 (20.0)	2 (20.0)	2 (16.7)	2 (16.7)
Hypokalaemia	0	0	0	0	1 (8.3)	1 (8.3)
Rash macular	0	0	1 (10.0)	1 (10.0)	1 (8.3)	0
Rash papular	0	0	1 (10.0)	1 (10.0)	0	0
Anaemia	1 (8.3)	0	1 (10.0)	0	1 (8.3)	1 (8.3)
Pneumonia	0	0	0	0	1 (8.3)	1 (8.3)
Abdominal pain	0	0	0	0	1 (8.3)	1 (8.3)
Skin discolouration	0	0	0	0	1 (8.3)	1 (8.3)

^a^Includes all AEs with onset on Day 11–14 post-dose for patients who were fed according to the protocol

^b^AEs with onset from Day 15 up to 28 days after the date of the last dose of AZD5363; includes AEs with onset before Day 15 that are not captured in the other two columns

^c^As assessed by the investigator

Elevations in plasma glucose (with associated increases in plasma insulin and C-peptide) were observed in the majority of patients dosed with AZD5363 in Parts A and B (Fig. 3b). There was no indication of glucose elevation in association with AZD5363 administration during the fasted period (up to 4 h post-dose). Taking into consideration the small patient numbers and the inter-patient rather than intra-patient comparison, the degree of glucose elevation in the fed state (Part B) was similar to that in the partially fasted state (Part A), but the elevation occurred earlier when AZD5363 was administered in the fed state.

## Discussion

The primary aims of this two-part, Phase 1 study were to evaluate whether a new tablet formulation of AZD5363 was comparable in PK, safety and tolerability to the original capsule form and to explore potential differences between fasted and non-fasted (following a standard meal) administration in terms of the PK properties of AZD5363 tablet.

The study had a within-subject fixed-sequence design in each of the two parts, whereby each patient received AZD5363 480 mg bid as tablets (Week 1) and capsules (Week 2) in a 4/3 schedule (Part A) or as tablets in a fasted (Week 1) and fed (Week 2) state on the fourth day of the 4/3 schedule (Part B). PK assessments were made over the 0- to 12-h dosing interval after the morning dose on the fourth day of each week. Steady-state conditions were assumed to have been achieved on these days, based on the half life of AZD5363 [11]. Likewise, the 7-day period between PK assessments was considered sufficient not to introduce any important carryover effect and is thus not expected to have affected the PK evaluation on Day 11.

The primary tumour location did not appear to influence the systemic exposure to AZD5363. Other causes of between-subject variability in absorption or elimination capacity, such as genetic polymorphisms of key enzymes/transporters or other metabolic differences, are not expected to influence the primary analyses and interpretation of this study as the relative bioavailability (tablet versus capsule and fed versus fasting, respectively) was estimated by comparing the systemic exposure (AUC and *C*_max_) within each patient.

Findings from Part A showed that the PK exposures (AUCτ and *C*_max_) of the AZD5363 tablet and capsule formulations were comparable when administered in a partially fasted state, and that the median *t*_max_ was approximately 1 h earlier for the tablet than for the capsule. Faster absorption in vivo is consistent with the more rapid dissolution of the tablet formulation observed in vitro (unpublished data). The results from Part B showed that the extent of exposure was comparable when the 480 mg dose was given as a tablet after an overnight fast and after the test meal (AUCτ fed:fasted ratio 0.89; 90% CI 0.76–1.05). The absorption was, however, delayed, as indicated from the lag time in the plasma concentration–time profile, which is in agreement with delayed gastric emptying after food and the biopharmaceutical properties of AZD5363. Once AZD5363 has been emptied into the small intestine, its absorption appears to be relatively rapid and similar to that after an overnight or a partial fast. The delayed, and potentially slower, absorption after food resulted in delayed *t*_max_ (median *t*_max_ of 2.0 versus 0.6 h) and lower *C*_max_ (geometric least-squares mean ratio 0.67; 90% CI 0.55–0.82). The following potential limitations to the accuracy of the PK parameter estimation have been identified: (i) the delayed absorption identified in the fed state may have resulted in an underestimation of *C*_max_, and to a lesser extent, AUCτ, because the frequency of sampling was lower at the later apparent *t*_max_ in the fed state; and (ii) *C*_max_ and AUCτ might be slightly biased because AZD5363 was to be administered in a partially fasted state on Days 1–3, but after an overnight fast, or with food, only on the fourth day of the 4/3 schedule. It can, however, be assumed that the potential bias is small and that the standard meal had no major effect on the bioavailability of AZD5363.

The intra-subject variability in AUCτ and *C*_max_ was moderate (around 20% CV), as predicted or lower than anticipated for the sample size calculation, and generally lower than the inter-subject variability. This supports the results having adequate precision and randomized crossover or fixed-sequence designs being preferable to parallel-group designs for PK comparisons in potential future studies. The observed PK exposure of AZD5363 at steady state (*C*_max_ and AUCτ) in this study was as expected based on results from an ongoing Phase 1 study of AZD5363 (Study D3610C00001) and in vitro dissolution data [11]. AZD5363 exposures in this study with AZD5363 tablet dosing in the 4/3 schedule were similar to exposures previously observed to correlate with tumour regression in preclinical models [11].

The safety and tolerability profile was not observed to be different between the tablet and the capsule formulations and appeared to be consistent with the emerging safety profile for AZD5363 as a capsule derived from Study 1 (NCT01226316) and Study 4 (NCT01353781). The safety and tolerability of the tablet did not appear to be different between the fed and fasted states and again reflected the emerging safety profile of AZD5363. Overall, safety observations of hyperglycaemia, diarrhoea and rash in this study are generally in agreement with the known safety profile of AZD5363 [11, 13, 14] and reflect reported effects of AKT inhibition [18, 19]. However, patient numbers were small; therefore, the safety and tolerability data should be interpreted with caution.

In summary, the results from this study demonstrated comparable PK properties of the AZD5363 tablet and capsule, as well as comparable bioavailability for the tablet given after an overnight fast and with food. The tablet formulation had slower absorption in the presence of food, resulting in lower and later peak concentrations. The clinical relevance of this food effect is currently unknown, hence a conservative approach has been taken; the recommendation is to administer the AZD5363 tablet formulation on an empty stomach, where possible, until further information becomes available.

## References

[1] Lindsley CW (2010). The Akt/PKB family of protein kinases: a review of small molecule inhibitors and progress towards target validation: a 2009 update. Curr Top Med Chem.

[2] Hennessy BT, Smith DL, Ram PT, Lu Y, Mills GB (2005). Exploiting the PI3K/AKT pathway for cancer drug discovery. Nat Rev Drug Discov.

[3] Liu P, Cheng H, Roberts TM, Zhao JJ (2009). Targeting the phosphoinositide 3-kinase pathway in cancer. Nat Rev Drug Discov.

[4] Bellacosa A, Kumar CC, Di Cristofano A, Testa JR (2005). Activation of AKT kinases in cancer: implications for therapeutic targeting. Adv Cancer Res.

[5] Altomare DA, Testa JR (2005). Perturbations of the AKT signaling pathway in human cancer. Oncogene.

[6] Davies BR, Greenwood H, Dudley P, Crafter C, Yu DH, Zhang J, Li J, Gao B, Ji Q, Maynard J, Ricketts SA, Cross D, Cosulich S, Chresta CC, Page K, Yates J, Lane C, Watson R, Luke R, Ogilvie D, Pass M (2012). Preclinical pharmacology of AZD5363, an inhibitor of AKT: pharmacodynamics, antitumor activity, and correlation of monotherapy activity with genetic background. Mol Cancer Ther.

[7] Addie M, Ballard P, Buttar D, Crafter C, Currie G, Davies BR, Debreczeni J, Dry H, Dudley P, Greenwood R, Johnson PD, Kettle JG, Lane C, Lamont G, Leach A, Luke RW, Morris J, Ogilvie D, Page K, Pass M, Pearson S, Ruston L (2013). Discovery of 4-amino-N-[(1S)-1-(4-chlorophenyl)-3-hydroxypropyl]-1-(7H-pyrrolo[2,3-d]pyrimidin-4-yl)piperidine-4-carboxamide (AZD5363), an orally bioavailable, potent inhibitor of Akt kinases. J Med Chem.

[8] Li J, Davies BR, Han S, Zhou M, Bai Y, Zhang J, Xu Y, Tang L, Wang H, Liu YJ, Yin X, Ji Q, Yu DH (2013). The AKT inhibitor AZD5363 is selectively active in PI3KCA mutant gastric cancer, and sensitizes a patient-derived gastric cancer xenograft model with PTEN loss to Taxotere. J Transl Med.

[9] Maynard J, Ricketts SA, Gendrin C, Dudley P, Davies BR (2013). 2-Deoxy-2-[18F]fluoro-D-glucose positron emission tomography demonstrates target inhibition with the potential to predict anti-tumour activity following treatment with the AKT inhibitor AZD5363. Mol Imaging Biol.

[10] Toren P, Kim S, Cordonnier T, Crafter C, Davies BR, Fazli L, Gleave ME, Zoubeidi A (2015). Combination AZD5363 with enzalutamide significantly delays enzalutamide-resistant prostate cancer in preclinical models. Eur Urol.

[11] Banerji U, Dean EJ, Pérez-Fidalgo JA, Batist G, Bedard PL, You B, Westin SN, Kabos P, Garrett MD, Tall M, Ambrose H, Barrett JC, Carr TH, Cheung SYA, Corcoran C, Cullberg M, Davies BR, de Bruin EC, Elvin P, Foxley A, Lawrence P, Lindemann JPO, Maudsley R, Pass M, Rowlands V et al (2017) A Phase 1 open-label study to identify a dosing regimen of the pan-AKT inhibitor AZD5363 for evaluation in solid tumors and in *PIK3CA*-mutated breast and gynecologic cancers. Clin Cancer Res (**Epub ahead of print**) 10.1158/1078-0432.CCR-17-2260 29066505

[12] Michalarea V, Lorente D, Lopez J, Carreira S, Hassam H, Parmar M, Sathiyayogan N, Turner A, Hall E, Serrano Fandos S, Seeramreddi S, Decordova S, Swales K, Ruddle R, Raynaud F, Tunariu N, Attard G, Molife LR, Banerji U, Plummer R, de Bono JS, Yap TA (2015) Accelerated Phase I trial of two schedules of the combination of the PARP inhibitor olaparib and AKT inhibitor AZD5363 using a novel intrapatient dose escalation design in advanced cancer patients. Annual Meeting of the Americal Association for Cancer Research. Philadelphia, PA, USA, 18–22 April:abst CT323

[13] Elvin P, Palmer A, Womack C, Tall M, Swales KE, Garrett MD, Banerji U, Tamura K, Cheung SYA, Lawrence P, Lindemann J, Ambrose H, Stephens C, Davies B, Foxley A, Pass M, Harrington EA, Barrett C (2014). Pharmacodynamic activity of the AKT inhibitor AZD5363 in patients with advanced solid tumors. J Clin Oncol.

[14] Esaki T, Seto T, Hirai F, Arita S, Toyokawa G, Hashimoto J, Tanabe Y, Kodaira M, Yonemori K, Hoshino Y, Yamamoto H, Kawata T, Lindemann J, Tamura K (2014). A phase I study to assess the safety and tolerability of the selective Akt inhibitor AZD5363 in Japanese patients with advanced solid tumours. Ann Oncol.

[15] AstraZeneca. Global Policy: Bioethics. (2015) Available at: http://www.astrazeneca.com/Responsibility/Code-policies-standards/Our-global-policies

[16] European Medicines Agency. Guideline on bioanalytical method validation. (2011). Available at: http://www.ema.europa.eu/docs/en_GB/document_library/Scientific_guideline/2011/08/WC500109686.pdf 10.4155/bio.12.44 22533559

[17] FDA. Guidance for industry: bioanalytical method validation. (2001). Available at: https://www.fda.gov/downloads/Drugs/GuidanceComplianceRegulatoryInformation/Guidances/ucm070107.pdf

[18] Chia S, Gandhi S, Joy AA, Edwards S, Gorr M, Hopkins S, Kondejewski J, Ayoub JP, Califaretti N, Rayson D, Dent SF (2015). Novel agents and associated toxicities of inhibitors of the pi3k/Akt/mtor pathway for the treatment of breast cancer. Curr Oncol.

[19] Spencer A, Yoon SS, Harrison SJ, Morris SR, Smith DA, Brigandi RA, Gauvin J, Kumar R, Opalinska JB, Chen C (2014). The novel AKT inhibitor afuresertib shows favorable safety, pharmacokinetics, and clinical activity in multiple myeloma. Blood.

